# Changes in Vegetable Consumption in Times of COVID-19—First Findings From an International Civil Science Project

**DOI:** 10.3389/fnut.2021.686786

**Published:** 2021-08-17

**Authors:** Irmgard Jordan, Gudrun B. Keding, Lena Stosius, Iwona Hawrysz, Katarzyna Janiszewska, Eleonore A. Heil

**Affiliations:** ^1^Center for International Development and Environmental Research, Justus Liebig University Giessen, Giessen, Germany; ^2^Division of Quality of Plant Products, Department of Crop Sciences, Georg-August-Universität Göttingen, Göttingen, Germany; ^3^Department of Human Nutrition, University of Warmia and Mazury in Olsztyn, Olsztyn, Poland; ^4^Working Group Nutrition Ecology, Faculty 09-Agricultural Sciences, Nutritional Sciences and Environmental Management, Justus Liebig University Giessen, Giessen, Germany

**Keywords:** COVID-19, vegetable diversity, income region, food intake, vegetable group, dietary diversity

## Abstract

The crisis related to the COVID-19 pandemic influenced food security and nutrition through both direct and indirect pathways. This ranged from short-term to long-term impacts, not only on health but also on food systems and thus on nutrition. This study aimed to identify how the observed constraints affected the food intake of populations across the globe. Here, special attention was paid to the consumption of vegetables and legumes and the diversity within these food groups. An online survey on Food and COVID-19 was conducted using a semi-structured questionnaire translated into several languages. Binary logistic regression models and Poisson regression models were calculated to evaluate changes in consumption patterns and to test potential determinants for the changes. For more detailed information on reasons for changes open ended questions were analysed qualitatively. Time spend at home, working from home, and mental stress were important drivers for changes in dietary intake according to the 1,042 respondents included in this analysis. The participants observed a change in food quantity (38%) and vegetable intake (27%). No changes were observed for the number of vegetable groups consumed, while significant reductions in diversity were detected within all vegetable groups. Moreover, associations between the number of consumed vegetable types during the COVID-19 pandemic and income regions as well as gender were found. The regression analysis showed that the level of decrease in vegetable diversity in the different vegetable groups were often depending on educational and occupational status, gender and household environment. Changes in food prices were related to changes in vegetable intake *per se*, overall vegetable diversity, and diversity within the provitamin A rich vegetable group. Food systems are not static and are transitioning quickly as could be observed during the Covid-19 pandemic. There is a need for a nutrition strategy to strengthen the resilience of vulnerable households to consume a diverse diet in adequate amount even in times of a pandemic.

## Introduction

COVID-19 (coronavirus disease 2019) refers to an infectious respiratory disease transmitted by SARS-CoV-II, which was first reported in Wuhan, China, in December 2019. Since then it was reported around the globe and was declared a global pandemic by the World Health Organisation (WHO) on 11th March 2020 (1, 2). By February 2021, globally more than 113 million cases and more than 2.5 million deaths were recorded. Globally, most cases were officially reported in the Americas and Europe (3).

Since COVID-19 has been declared a global pandemic, countries all over the world took measures such as contact and travel restrictions, store closures, curfews during day or at night or other general confinements to limit the further spread of the virus. These restrictions were in turn affecting the economic situation of many people and thus the purchasing power of these households (4).

The crisis related to the COVID-19 pandemic influenced food security and nutrition through both direct and indirect pathways. Direct pathways may be trade and transport restrictions which negatively impacted on food availability whereas indirect pathways include effects like no school feeding due to school closures or loss of income and thus reduced food purchasing power. This ranged from short-term to long-term impacts, not only on health but also on food systems and thus on nutrition. In this context, the High level of Panel Experts of the Committee on World Food Security (CFS) have emphasised that the initial situation of individual countries and regions and their resilience to such crises will play a decisive role in determining the severity of the disruption as the pandemic evolves (5).

The pandemic challenged the economic and physical access to sufficient and nutritious food, especially for already vulnerable groups and countries (6). Because of trade restrictions and panic buying food items run out of stock or were not affordable for low income households after food prices raised following the trade restrictions (7–9). In countries where workers for food production needed to be hired from other countries food production and processing were affected, too, reducing the availability of perishable foods and subsequently rising prices (9, 10). The impact on the food chain, in the form of restaurant closures and supermarket regulations to avoid food shortages, became apparent soon after first countries started with border closures to reduce the risk of transmitting the virus due to high mobility (9). The services provided by canteens at the workplace, in schools and universities were also minimised or completely cut off which put especially children worldwide at risk to become food insecure (11, 12). Families with school children who relied on meals provided at school struggled to feed their children properly. Although preventive measures were being taken, social media constantly shows out-of-control situations in food stores, which can lead to food shortages and the faster spread of the virus (13). Adjustments were also being implemented to protect and serve citizens and to support the food sector, including delivery services, e.g., school meals were brought to the children's homes (UK) (14), fast food companies to provide school meals (Spain) (15), food baskets were offered from balconies (Naples, Italy) (16) or placed at fences (Berlin, Germany) (17), or food aid was distributed by the Governments (Uganda and USA) (18, 19). In Brazil this concept was shown to work well in large cities, but its accessibility did not reach all socioeconomic groups and geographic locations (13).

Before the Covid-19 pandemic began, a group of scientist looked at how current dietary practises impact planetary health (20). The same group called for substantial transformations in food production and consumption to benefit human and environmental health. This would require among others a shift toward healthy dietary patterns, i.e., limited intake of animal source foods, and an increase in the consumption of legumes, vegetables, fruits, nuts, and seeds (20). The World Health Organisation nutrition advice for adults during the Covid-19 outbreak emphasises the need to regular consume fruits, vegetables, and legumes (21, 22). However, the nutrient values of vegetables can vary thousandfold among different varieties of the same food (23). Many studies on Covid-19 and dietary changes looked at overall changes in food purchasing patterns, consumption and lifestyle only and paid little attention to dietary diversity or even diversity within a food group (6, 8, 9, 12, 24). The “Food systems in times of COVID19” (COVID-Food systems) project aimed to identify how the observed constraints affect the food systems and dietary behaviour of populations across the globe. The objective of this study was to investigate changes in food intake following up on the trade restrictions and recommendations along the debate on planetary health. Following up on the WHO recommendations to consume adequate amount of vegetables and legumes to maintain health, special attention was paid to the change in the consumption of vegetables and legumes and its diversity.

This paper thus presents the analysis of the dietary changes (food quantity, overall vegetable consumption, and vegetable diversity) in relation to the restrictions and lockdown scenarios in diverse populations. Furthermore, individual and environmental characteristics as a possible cause of these changes were investigated to describe the groups most vulnerable to greatest reduction in diversity in vegetable consumption.

## Method

In close collaboration with members of the international research community from 12 different countries who were interested to join the COVID-Food systems project we developed an online semi-structured questionnaire. The transdisciplinary developed questionnaire asked for socio-demographic information, living environment of the participants, aspects of the participants food systems, food intake, and aid programs as well as the participants perceptions toward changes following the restrictions established in the respective countries. After a consensual validation process including two rounds of pre-testing the questionnaire consisted finally of 65 questions of which 15 were closed, 15 were open-ended, while 35 were designed as mixed questions. The closed-ended questions offered a list of predetermined responses. The open ended questions asked for observations made by the participants providing space for a text without limitation of characters. The mixed questions were offering space to comment and add information about the responses made to the question which had offered a predetermined response scale, e.g., “yes/no/don't know or other, please specify.” Changes in food consumption were assessed retrospectively using the same questions to assess the situations prior to and since the pandemic started. The question on price change was measured using a Likert scale (strong increase, little increase, no change, little decrease, and strong decrease).

Various translations of the questionnaire, originally designed in English were developed during the ongoing data collection, namely into Chinese, German, Polish, Russian, Spanish, and Vietnamese among others. The translations were back-translated to English for validation of the translation. The survey took place over a period of 4.5 months starting on 17.04.2020 with the English version.

There were no exclusion criteria for participants. The questionnaire was accessible to anyone with a device with internet access; resulting in a convenience sampling. The SoSci Survey platform was used to create and conduct the online survey tool (25). Data protection was done in accordance with the German data protection laws and regulations, the survey server and operator were placed in Munich, Germany. The survey platform is free for non-commercial research like this study. The authors do not have any conflict of interest.

The link to the survey was uploaded on the project website on “Sustainable Food systems—going beyond Food Security” and on the institutional website of the Centre for International Development and Environmental Research of the University of Giessen (26, 27). The survey was promoted by all questionnaire developers from 12 different countries with different impact *via* social media, email lists, personal contacts and their networks, and the email distribution list of the University of Giessen. The participation was voluntary at any stage and the participants had to actively confirm their willingness to participate. Therefore, no institutional approval was needed according to the review board of the Justus-Liebig University Gießen, Germany.

In total, the link to the survey was used 7,566 times. The klicks included any use of the link whether it was done on purpose or by accident or by a search engine. Thus, no conclusions can be made on how many people were interested in the study but rejected their participation after reading the introduction. Out of the total klicks, 1,528 were counted as completed questionnaires (participants responded to the final question). Participants who did not confirm their willingness to participate were excluded in the data analysis.

As we assume that the data has a risk to be blurred we used an exploratory approach for the analysis. Consequently, we did not follow a specific hypothesis and abstained to do a sample size calculation prior to the data collection.

### Selection of Data Included Into This Study

**Figure 1:**
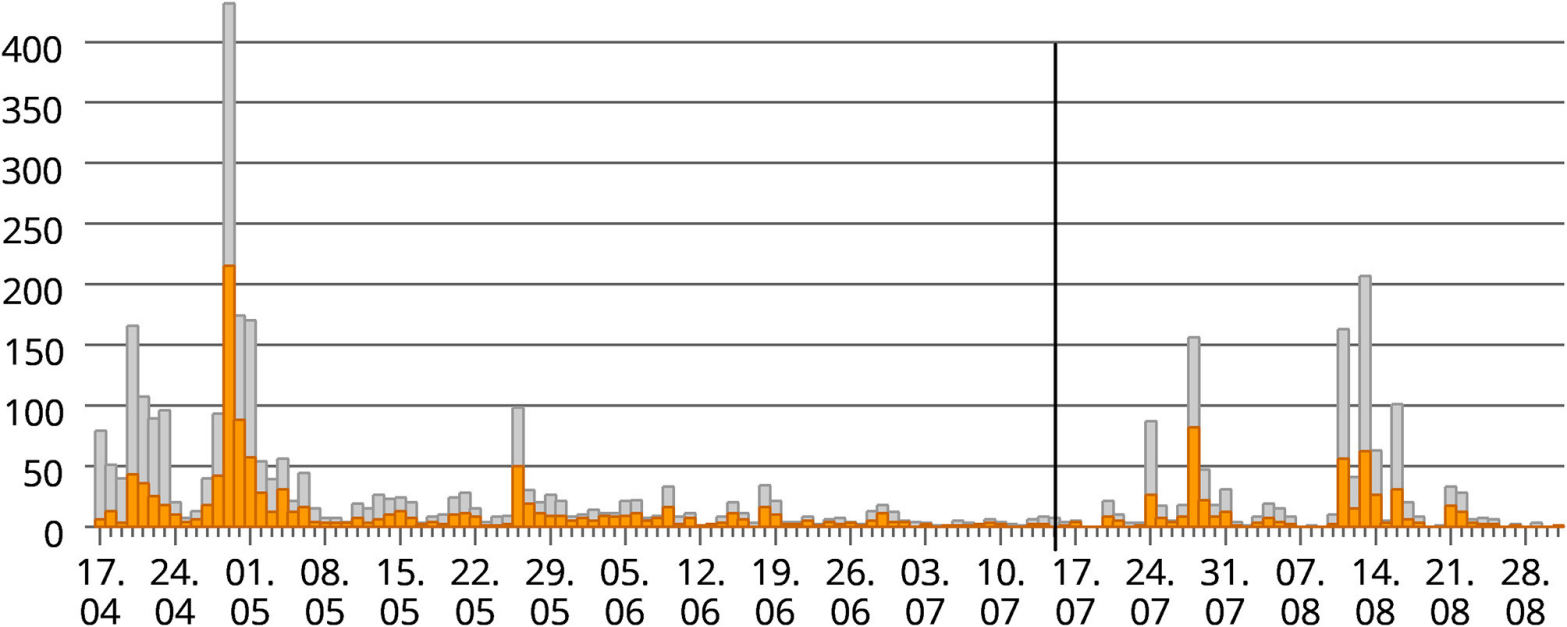
Response pattern of the data collection over time (17.4.-31.8.2020) (x-axis, date of data collection; y-axis, number of responses; orange, completed questionnaires; grey, total responses but incomplete or klicks; black vertical line, end of data collection for this study).

There were several peaks in responses following the various promotion campaigns. The completed responses came from individuals living in 62 different countries. Mid July 2020 a special data collection campaign was started in Poland which resulted in a series of new responses from Polish citizens (Figure 1). To avoid confusion with the first lockdown/restriction wave it was decided to exclude for this study the data which was collected after the 15.7.2020.

### Preparations for Data Analysis

The responses given in the option “other” in the responses to questions related to *household members, education, occupation, housing environment*, and *restrictions*, were integrated into the existing response categories or new response categories were created if necessary. In order to show regional differences, countries were grouped according to their income status following the classification of the World Bank based on the gross national income (GNI) per capita; the upper cut offs were set at US$1,035 for low income countries, US$4,045 for lower middle income countries, and US$12,535 for upper middle income countries (28).

#### Food Quantity

Respondents were asked to evaluate the amount of food they were eating at the time they filled the questionnaire in comparison to the before-pandemic time. Three answers were available: (a) just as much than before the pandemic, (b) less than before the pandemic, and (c) more than before the pandemic.

Binary logistic regression models were used to calculate Odds Ratios (OR) for the change. To obtain more precise information on the amount of food, food items, and reasons for changes the open-ended parts of the question were evaluated qualitatively based on summaries of the provided responses. All comments given in any other language than English were translated to English. Quoted comments were corrected for spelling mistakes. Country of residence, age, and gender were indicated in parentheses for each direct quote given in quotation marks. In the case of indirect quotes only the country of residence was reported in parentheses.

#### Vegetable Consumption and Vegetable Diversity

The impact of COVID-19 pandemic on the consumption of vegetables was evaluated based on the respective questions (a) “Prior to Covid-19 pandemic: Did you consume any of the vegetables listed below over a period of 4 weeks (1 month) prior to Covid-19?” and (b) “Since the pandemic started: Did you consume any of the vegetables listed below in the last 4 weeks?”. It was distinguished between the periods of time the change occurred looking at 4, 8, and 12 weeks retrospectively starting at the time of the interview. A list of 96 types of vegetables was stratified into 5 groups: *dark green leafy vegetables* (e.g., amaranthus leaves, Feldsalat, bok choy)*, vitamin-A rich vegetables* (e.g., carrots, pumpkin, sweet red pepper)*, starchy vegetables* (e.g., cassava, white potatoes, corn/maize)*, legumes* (adzuki beans, chickpeas, sweet peas)*, other vegetables* (e.g., tomatoes, asparagus, cabbage). The groups were defined based on guidelines for the vegetable groups used to estimate the minimum dietary diversity for women (29). Data without time reference were not considered. Vegetable diversity was defined counting the (a) number of groups covered in the diet and (b) number of different types consumed within each vegetable group prior to and since the pandemic. The numbers counted for the time since COVID-19 were subtracted from the number prior to the pandemic, thus, positive values indicate a reduction and negative values an increase in diversity over time.

#### Food Prices

To analyse if perceived changes in food prices had an influence on the amount of food, vegetable consumption and vegetable diversity consumed, a price index was calculated. A perceived price change was assessed based on the 10 food groups of the minimum dietary diversity score for women (staple foods, legumes, nuts and seeds, milk and milk products, meat and fish, eggs, dark green leafy vegetables, vitamin-A rich vegetables and fruits, other vegetables, other fruits) (29). The changes in prices for all food groups were summed up with 2 points for a “strong increase,” 1 point for a “little increase,” 0 points for “no change,” −1 points for a “little decrease,” and −2 points for a “strong decrease” per food group.

### Statistical Analysis

Binary logistic regression models were used to analyse which factors have an influence on changes in food intake. A Poisson regression was calculated for food intake and in particular vegetable diversity to determine the time effect. Estimated marginal means are presented to visualise effects. Binary logistic regression and Poisson regression models were also calculated and adjusted for age, gender, and income regions in order to test the effects of lockdown and restriction scenarios. The models were created with the procedure Genlinmixed in SPSS and robust standard errors were used. Control variables were listed below the tables presenting our findings. The *p*-values of the multiple pairwise comparisons in the regressions were calculated according to sequential Bonferroni. IBM SPSS Statistics 27 was used for all statistical analysis.

## Results

**Figure 2:**
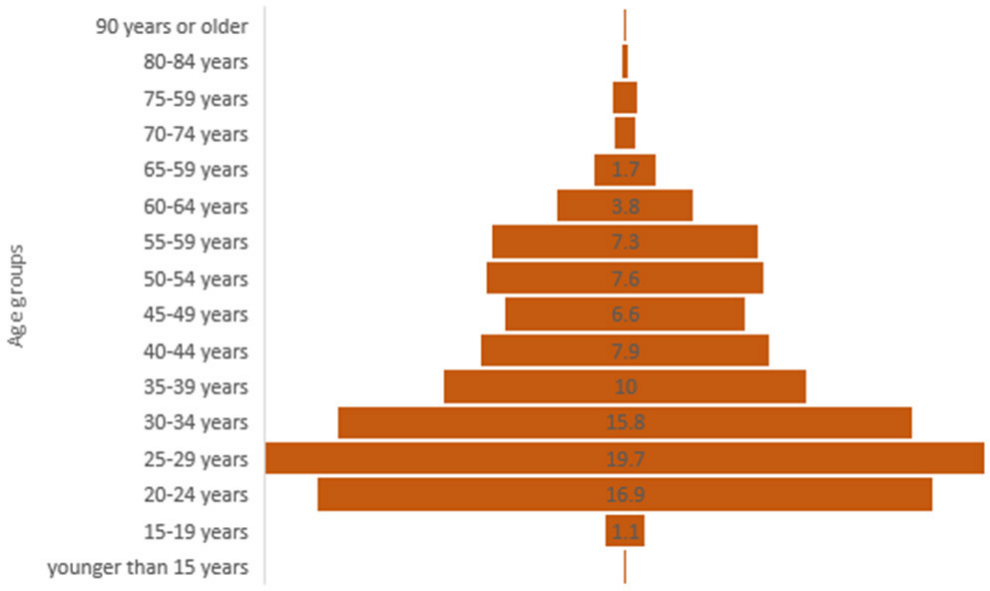
Distribution of age groups within the study population indicated in percent (*n* = 1,041).

During the survey period 17.4.-15.7.2020, 1,083 participants completed the questionnaire, of whom 1,042 gave their consent that the data from the questionnaire may be used for research purposes. More than 3/4 of the participants were females (77%), while 22% were male and 0.7% responded as non-binary. Two thirds of the participants (62%) were between 20 and 39 years old. The group younger than 15 and all groups from the age of 70 and above accounted for <1% each (Figure 2).

More general characteristics are presented in Table 1. Most of the respondents were university graduates (71%), followed by high school graduates or people with an A-levels certificate (15%). Being an employee, a civil servant and University student or training was the most mentioned occupation (29, 28, and 28%, respectively).

**Table 1:** General characteristics of the participants.

	**Percentage**
**Gender (** ***n*** **=** **1,042)**	
Female	76.7
Male	22.3
Non-binary	0.7
Preferred not to say	0.3
**Educational level (** ***n*** **=** **1,041)**	
No degree or below the level of high school	5.6
Finished high school	14.8
Completed apprenticeship or vocational baccalaureate diploma	8.9
University degree	70.7
**Occupation (** ***n*** **=** **1,035)**	
Student in school	2.0
University student or training	27.7
Unemployed	3.7
Employee	28.8
Self-employed	7.2
Civil servant	27.7
Retirement/Pension	2.8
**Geographical region (** ***n*** **=** **1,037)**	
Asia and the Pacific	14.8
Latin America and the Caribbean	4.5
North America	3.8
Africa	4.2
Europe	72.7
**Income region (** ***n*** **=** **1,037)**	
Low income countries	1.5
Lower middle income countries	11.9
Upper middle income countries	9.1
High income countries	77.5
**Living environment (** ***n*** **=** **1,039)**	
Rural area	21.4
Peri urban area	14.0
Small town (<1 h walking distance from farmland)	16.8
Small town (1–4 h walking distance from farmland)	10.9
Big town (1–4 h walking distance from farmland)	3.5
Big town (province capital)	11.8
City	10.1
Mega city	2.8
Capital city	8.8
**Household types: “I live … (** ***n*** **=** **1,026)**	
Alone	15.6
With my partner	29.6
2 generation family	18.3
3 generation family	5.7
1 generation shared flat	14.3
2 generation shared flat	7.6
Single parents with children of different age	1.9
Other	1.4
Different family types with children of unknown age	5.6
**Lockdown scenarios (multiple responses) (** ***n*** **=** **1,042)**	
Contact restrictions	72.6
Travel restrictions	74.8
Only food retailers/supermarkets, drugstores and pharmacies are open	53.1
Curfew during day	4.8
Curfew at night	6.8
You are not allowed to leave your house but only to buy food	11.7
Other restrictions	10.7
Not that I know	3.5
**Change in food quantity since the pandemic started (** ***n*** **=** **1,037)**	
Eat less food (any)	15.1
Amount of food (any) did not change	62.0
Eat more food (any)	22.9

*Estimated mean age = 37 years; estimated based on age group prevalences (Figure 2)*.

The majority (91%) reported no change in the occupational status due to COVID-19, 4% claimed that their working hours had decreased, their job had been temporarily suspended or they had experienced economic losses due to COVID-19 which might affect the level of food expenditure. Still, loss of their jobs due to the pandemic was reported by 5% of the respondents. Any support from the government, associations, religious communities, or individuals was received by 8%.

Overall, 62 countries were covered in this study, but the countries were unevenly represented. Nearly all geographical regions were covered, however the majority of the respondents resided in Europe (73%) at the time of their participation. The majority of participants lived in Germany (67%), followed by Vietnam (8%), China (4%), USA (4%), Colombia (1.4%), Poland (1.3%), and Kenya (1.3%); the remaining 14% of the respondents live in 53 different countries (Supplementary Table 1). Stratified by income region, 77.5% lived in high income countries, 9.1% in upper middle income countries, 11.9% in lower middle income countries, and only 1.5% in low income countries. In the course of the survey, 63% reported experiencing a lockdown and 16% were not affected anymore while 21% stated that they had not been affected at all by a lockdown. Table 1 shows that contact and travel restrictions (73 and 75%) as well as restricted store openings (53%) were predominant. Curfews during day, curfews at night and full lockdowns only affected 4.8, 6.8, and 11.7% of the respondents, respectively. Only 3.5% of the participants reported that they did not know about any existing restrictions in their living area.

### Changes in Food Quantity

The majority of the respondents (62%) did not observe any change in the amount of food they consumed. The proportion of people who observed an increase over time was higher than those who observed a decrease (23 vs. 15%) (Table 1). The greatest change in food quantity occurred in the group of people who indicated that they had entered into a lockdown. The change occurred in both directions: decrease and increase in the amount of food consumed. Compared to the group that was no longer affected by a lockdown, the ones that did not experience a lockdown indicated more often to eat less than before the pandemic (96 and 13%, respectively). The share of respondents eating more than before the pandemic ranged between 20 and 24% regardless of whether they (had) experienced any lockdown or not (Table 2).

**Table 2:** Perceived change in food quantity in relation to lockdown scenarios indicated in percent in reference to prior to the pandemic.

	**Eat less food**	**Amount of food did not change**	**Eat more food**	Change in vegetable intake^*^
Low income countries *N* = 16	25.0	68.8	6.3	28.6
Lower middle income countries *N* = 123	19.5	63.4	17.1	21.1
Upper middle income countries *N* = 94	18.1	64.9	17.0	23.3
High income countries *N* = 804	14.0	61.1	24.9	28.6
No lockdown (*N* = 215)	12.6	67.4	20.0	22.7
No lockdown anymore (*N* = 163)	8.6	68.5	22.8	26.5
Lockdown (*N* = 655)	17.4	58.7	23.9	28.9

* *Perceived change, either decrease or increase*.

To obtain more information about the possible reasons for a change we asked the respondents to give a more detailed explanation for reported change in food intake. Most frequently mentioned reasons for an increase in the consumed amount of food were isolation, boredom, more home-cooked meals, more free time, spending more time at home, working from home, having meals together with the family, and mental stress. All of these reasons might be direct or indirect result of the restrictions implemented by the governments.

The binary logistic regression on the decrease in the amount of food eaten confirmed a significant influence of the lockdown scenarios. The proportion of people who ate less in the group that experienced a lockdown was higher than in the group that was no longer in lockdown, with an average difference of 14.2% (−0.142, 95% CI [−0.257, −0.027], *p* = 0.010). No significant effects could be identified for the specific types of restrictions and in regard to increase in the amount of food and the various restrictions and lockdown scenarios (Table 3).

**Table 3:** Odds ratios for perceived changes in food and vegetable intake.

	**Food quantity ^§^**		**Vegetable intake^§^**	Vegetable categories ^*^^#^	**Vegetable diversity^#^**				
	**Decrease**	**Increase**			**Dark green leafy**	**Provitamin A rich**	**Starchy**	**Legumes**	**Other**
**Basic model, not adjusted**									
OR					**0.832**	**0.866**	**0.878**	**0.848**	**0.938**
*p*					**0.000**	**0.000**	**0.000**	**0.000**	**0.000**
95% CI					**0.805/0.860**	**0.841/0.893**	**0.836/0.923**	**0.817/0.879**	**0.909/0.967**
**No lockdown-no lockdown anymore**									
OR	0.547	1.038	1.019	1.387	**1.191**	0.981	1.072	1.057	1.109
*p*	0.102	0.892	0.476	0.216	**0.028**	0.779	0.543	0.552	0.158
95% CI	0.265/1.127	0.605/1.782	0.968/1.072	0.825/2.332	**1.019/1.392**	0.861/1.119	0.857/1.341	0.880/1.269	0.961/1.280
**No lockdown-lockdown**									
OR	1.315	1.133	1.036	1.349	1.126	1.083	1.161	1.107	1.040
*p*	0.310	0.587	0.091	0.167	0.061	0.104	0.123	0.172	0.493
95% CI	0.775/2.233	0.722/1.776	0.994/1.080	0.882/2.064	0.994/1.275	0.984/1.192	0.960/1.404	0.957/1.281	0.930/1.163
**No contact restrictions-contact restrictions**									
OR	1.063	0.977	0.768	1.033	1.030	1.067	1.135	1.068	1.079
*p*	0.804	0.907	0.171	0.106	0.598	0.173	0.140	0.344	0.156
95% CI	0.654/1.728	0.656/1.453	0.527/1.120	0.993/1.075	0.924/1.147	0.972/1.171	0.959/1.343	0.932/1.224	0.971/1.198
**No travel restrictions-travel restrictions**									
OR	0.998	0.852	0.859	**0.968**	1.020	0.947	0.912	0.954	0.949
*p*	0.992	0.399	0.409	**0.037**	0.698	0.191	0.216	0.436	0.277
95% CI	0.625/1.591	0.587/1.237	0.598/1.234	**0.939/0.998**	0.923/1.128	0.872/1.028	0.789/1.055	0.848/1.074	0.863/1.043
**No store closures-only food stores, drugstores and pharmacies are open**									
OR	1.230	1.351	1.308	0.991	0.993	1.023	0.982	1.059	1.046
*p*	0.296	0.068	0.083	0.535	0.870	0.524	0.766	0.285	0.273
95% CI	0.833/1.816	0.978/1.866	0.965/1.773	0.964/1.019	0.915/1.079	0.953/1.098	0.871/1.107	0.953/1.177	0.965/1.132
**No curfew during day-curfew during day**									
OR	1.131	1.356	1.454	0.936	1.021	0.941	0.940	0.872	0.904
*p*	0.813	0.584	0.408	0.341	0.894	0.679	0.716	0.442	0.539
95% CI	0.408/3.135	0.455/4.038	0.598/3.536	0.815/1.074	0.755/1.379	0.706/1.256	0.671/1.315	0.614/1.238	0.654/1.249
**No curfew at night-curfew at night**									
OR	1.802	1.002	1.700	1.034	0.925	1.129	1.128	**1.707**	0.982
*p*	0.162	0.997	0.182	0.543	0.560	0.325	0.367	**0.000**	0.899
95% CI	0.788/4.122	0.371/2.704	0.780/3.704	0.929/1.152	0.710/1.204	0.886/1.438	0.868/1.468	**1.301/2.240**	0.739/1.305
**You are allowed to leave the house-you are not allowed to leave the house but only to buy food**									
OR	1.014	0.943	0.819	1.023	1.043	0.949	1.027	0.921	1.061
*p*	0.966	0.824	0.470	0.458	0.644	0.479	0.800	0.425	0.478
95% CI	0.541/1.900	0.559/1.590	0.475/1.410	0.963/1.086	0.871/1.249	0.820/1.098	0.834/1.264	0.751/1.128	0.901/1.248

§ *Binary logistic regressions calculated for the change*.

# *Poisson regressions calculated for the time period since the onset of COVID-19, significance level: p < 0.05, 95% CI = 95% confidence intervals, adjusted for age, gender, and income regions*.

* *Vegetable categories (max. 5 = dark green leafy vegetables, provitamin A rich vegetables, starchy vegetables, legumes, and other vegetables), dark green leafy vegetables (max. 18), provitamin A rich vegetables (max. 8), starchy vegetables (max. 9), legumes (max. 17), and other vegetables (max. 33)*.

*OR, odds ratio; CI, confidence interval. Bold means: values were estimated to be statistically significant, p < 0.005 and the respectives values were bolded to facilitate reading*.

Overall, the change in food quantity in relation to income regions was lowest in the low income countries and highest in the high income countries. The group of people who said they ate more than before the pandemic was represented most frequently in the high income countries (25%) and least frequently in the low income countries (6%). In contrast, the prevalence of participants reporting to eat less was lowest in high income countries (14%) and highest in low income countries (25%) (Table 2).

With an increase by one age-group (Figure 2), the chance to increase the amount of consumed food decreased by 9.6%, adjusted (ad) for gender, income region, occupation, education, household type and living environment (adOR = 0.904, 95% CI [0.831, 0.983], *p* = 0.018).

### Changes in Overall Vegetable Consumption

**Figure 3:**
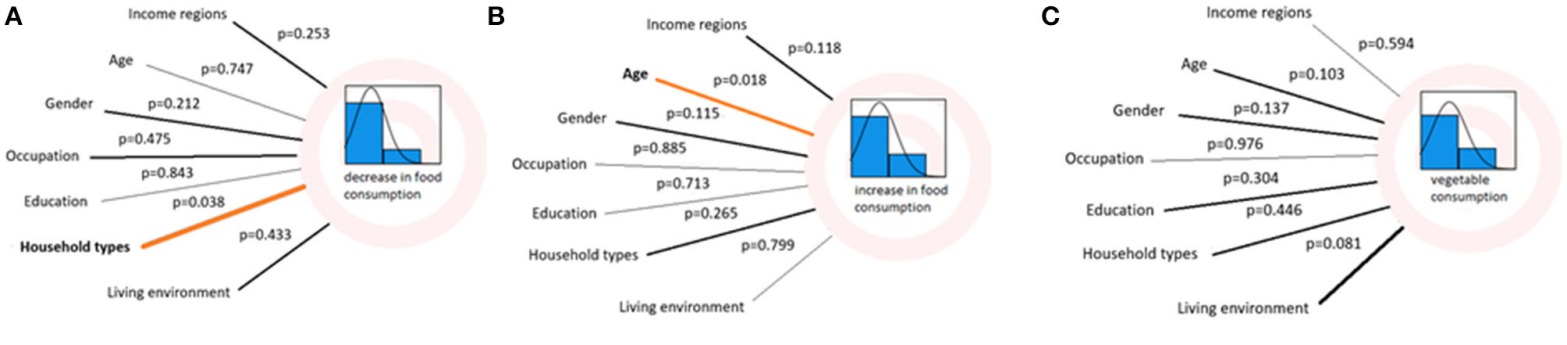
Results of binary logistic regression models for correlations between personal factors and housing situations and **(A)** decrease in food quantity, **(B)** increase in food quantity, **(C)** change in vegetable consumption (orange line indicates a correlation with *p* < 0.05).

Out of the 1,042 participants included in this study, 995 reported in detail on their vegetable consumption. Out of these, 27% indicated a change in their vegetable consumption which was not associated with age (Figure 3C). The proportion of participants who indicated a change decreased with the length of the time period (4, 8, and 12 weeks retrospectively starting at the time of the interview), ranging from 25% in the last 4 weeks to 8% in the last 12 weeks. Even though the overall effect of “living environment” was not significantly influencing vegetable consumption (Figure 3), the pairwise comparisons between mega cities and peri urban areas as well as mega cities and small towns (farmland within 1 h walking distance) showed significant differences with 21% more respondents from the peri urban area and 20% more respondents from the small town reporting a change in their vegetable intake compared to respondents from mega cities (0.207, 95% CI [0.007, 0.407], *p* = 0.033 and 0.200, 95% CI [0.001, 0.399], *p* = 0.047, respectively).

The change in vegetable consumption occurred in both directions: increase and decrease which resulted in an overall “no change” for all respondents. Reasons for decrease were “reduced access and availability” as reported from Bangladesh, Ecuador, Guatemala, Ireland, Kenya, New Zealand, Poland, Vietnam, Spain, Tanzania, and USA, “increased prices” reported from Ecuador, Fiji, Kenya, and Germany or because respondent went “less shopping” (Germany and USA) or “those who provided the meals, do not make balanced dishes and you have to eat what they are offering” as mentioned by a respondent from Columbia (35–39 year old woman). “Children do not eat as diversely” or “my parents buy less vegetables than I would” were mentioned by women from Germany (35–39 years old and 20–24 years old, respectively) indicating new household settings due to students staying at home. But also, time constraints and stress were pointed out by a man as factor influencing vegetable consumption: “Less vegetables, [because] less time to cook (work and childcare), more emotional stress” (Germany, 45–49 years, male).

### Changes in Vegetable Diversity

The mean number of vegetable groups covered in the diets was 4.5 out of 5 for the two time points: prior to and since the pandemic started. No significant association was found between age and diversity within the observed vegetable groups excluding “other vegetables.” The latter was associated with a small increase over “time” by age (Supplementary Table 2). In the case of overall vegetable diversity, the consumption of vegetable categories showed a shift of the median only for low income countries. The medians for the number of different vegetable types consumed per each food group were more stable over time in the high income countries than in the other regions. The open responses indicated a trend from fresh vegetables to frozen, canned vegetables or storable vegetables. At the same time study participants reported that they have “more time to cook” (Poland and Germany) and that they “eat more carrots and reduce the total amount of vegetables” (China, 30–34 years, male) or increased their vegetable consumption “for better health and immunity” (Colombia, Ecuador, Ethiopia, Germany, Honduras, Vietnam). More vegetables were consumed also because “no fish or butchery are open” (India, 50–54 years, male) or “mainly due to the fact that stores run out of pasta” (Germany, 50–54 years, female).

The diversity of the “dark green leafy vegetable” consumption reduced since the beginning of the pandemic with an average decrease of 0.71 vegetable types (0.706, 95% CI [0.579, 0.832], max = 18 types, *p* < 0.001). The overall variety within the food group “provitamin A rich vegetables” ranged from 0 to 8. The diversity within the vegetable groups decreased on average by 0.39 vegetable types (0.389, 95% CI [0.308, 0.470], *p* < 0.001). With an average value of 1.2 before and 1.1 since COVID-19, “starchy vegetables” was the group of vegetables with the lowest variety (min-max: 0–9). Nevertheless, a decrease in diversity within the group was observed with an average decrease of 0.15 vegetable types (0.147, 95% CI [0.091, 0.203], *p* < 0.001). Between 0 and 17 legume types were counted in this study. The mean value was 3.4 before the pandemic and 2.9 since its beginning. Also, for “legumes,” a significant time effect was shown with an average decrease of 0.51 legume types since the onset of the pandemic (0.513, 95% CI [0.398, 0.628], *p* < 0.001). “Other vegetables” was the group with the highest variability. In total 33 different types of “other vegetables” were mentioned. The mean value was 8.7 before and 8.1 since COVID-19. Like for the other vegetable groups, the diversity decreased significantly since the beginning of the pandemic with an average decrease of 0.54 vegetable types (0.542, 95% CI [0.286, 0.798], *p* < 0.001). The actual number of different vegetable types consumed per vegetable group and the mean values prior to and since the pandemic started are presented in Supplementary Table 3.

The OR of the basic model estimated that individuals had a lower chance of eating a greater number of different “dark green leafy vegetables” (16.8%), “provitamin A rich vegetables” (13.4%), “starchy vegetables” (12.2%), “legumes” (15.2%), and “other vegetables” (6.2%) since the onset of the pandemic (calculated based on OR) (Table 3).

The Poisson regressions presented in Table 3 show that travel restrictions had a significant effect on vegetable diversity, thus, a 3.2% lower chance for consuming a higher number of vegetable types, when age, gender and income regions were hold constant (adOR = 0.968, 95% CI [0.939, 0.998], *p* = 0.004). Respondents that were no longer affected by a lockdown had about a 1.2 times higher chance of consuming a higher diversity of dark green leafy vegetables than those that had not been affected (adOR = 1.191, 95% CI [1.019, 1.392], *p* = 0.028). Respondents that experienced a curfew at night having about a 1.7 times higher chance for a more diversified legume intake compared to those that were not affected (adOR = 1.707, 95% CI [1.301, 2.240], *p* < 0.001). In contrast, affected by a curfew at night led to an increase in diversity of the legumes by 0.8 in times of COVID-19 (−0.813, 95% CI [−1.431, −0.194], *p* = 0.010). For all other restrictions and vegetable groups no significant correlations were found.

Reduction in vegetable diversity was associated in this study with “income region,” gender, education level, occupation, household type, and the living environment of the respondent. Hence, respondents living in lower middle income countries, being a woman, having a university degree, being unemployed, living in a 3-generational family and living in a small town were in general associated with the greatest reduction in diversity in each five vegetable groups. The most pronounced reductions were found for dark green leafy vegetables, legumes, and other vegetables, the lowest reductions in the vegetable groups “starchy vegetables” and “provitamin A rich vegetables” (Table 4). The Poisson model for dark green leafy vegetables showed, for example, a significant reduction in the diversity since the onset of the COVID-19 pandemic for all income regions with the greatest decrease occurring in the lower middle income countries [1.1 vegetable types less (1.076, 95% CI [0.542, 1.611] *p* < 0.001)]. Also, for both woman and man there was a significant reduction in the diversity of consumption of dark green vegetables with women consuming on average 1 vegetable type (1.001, 95% CI [0.540, 1.462], *p* < 0.001) and men 0.9 vegetable type (0.927, 95% CI [0.543, 1.311], *p* < 0.001) less than before COVID-19. The 3-generation families were the ones with the greatest reduction of diversity of dark green leafy vegetables since the outbreak of COVID-19 [1.3 vegetable types less (1.281, 95% CI [0.581, 1.980), *p* < 0.001)]. As for the living environments, inhabitants of cities had the greatest decrease in their diversity of dark green leafy vegetables (1.563, 95% CI [0.879, 2.247], *p* < 0.001).

**Table 4:** Results of Poisson regressions for changes since the onset of the pandemic in the diversity of vegetable categories, dark green leafy vegetables, and provitamin A rich vegetables.

	**Vegetable categories**			**Dark green leafy vegetables**			**Provitamin A rich vegetables**		
	**Mean difference**	***p***	**95% CI**	**Mean difference**	***p***	**95% CI**	**Mean difference**	***p***	**95% CI**
**Income regions**									
Low income	0.003	0.993	−0.644/0.650	**1.043**	**0.021**	**0.162/1.925**	0.027	0.822	−0.211/0.266
Lower middle income	0.180	0.140	−0.059/0.418	**1.076**	**0.000**	**0.542/1.611**	**0.369**	**0.013**	**0.079/0.658**
Upper middle income	0.060	0.697	−0.244/0.365	**0.678**	**0.006**	**0.199/1.157**	0.278	0.159	−0.109/0.666
High income	0.004	0.975	−0.230/0.237	**0.728**	**0.003**	**0.254/1.203**	0.103	0.438	−0.158/0.365
**Gender**									
Female	0.050	0.712	−0.215/0.315	**1.001**	**0.000**	**0.540/1.462**	0.227	0.058	−0.008/0.461
Male	0.078	0.536	−0.168/0.323	**0.927**	**0.000**	**0.543/1.311**	0.171	0.083	−0.022/0.365
**Education**									
No degree/degree below level of	0.173	0.412	−0.241/0.588	**1.284**	**0.000**	**0.574/1.994**	0.132	0.512	−0.263/0.527
high school									
High school/A-level degree	−0.004	0.975	−0.254/0.246	**0.891**	**0.000**	**0.462/1.320**	0.077	0.487	−0.141/0.295
Apprenticeship/vocational	0.041	0.774	−0.238/0.320	**0.670**	**0.004**	**0.210/1.129**	**0.275**	**0.044**	**0.007/0.543**
baccalaureate diploma									
Vocational university diploma	0.046	0.671	−0.167/0.260	**0.984**	**0.000**	**0.614/1.355**	**0.317**	**0.001**	**0.137/0.497**
**Occupation**									
Student in school	−0.047	0.885	−0.677/0.584	0.426	0.502	−0.830/1.683	−0.320	0.290	−0.914/0.273
University student/Trainee	0.189	0.101	−0.037/0.414	**0.929**	**0.000**	**0.541/1.318**	**0.310**	**0.012**	**0.069/0.551**
Unemployed	0.109	0.593	−0.292/0.510	**1.173**	**0.003**	**0.410/1.935**	**0.445**	**0.029**	**0.046/0.844**
Employee	0.105	0.378	−0.128/0.337	**1.109**	**0.000**	**0.730/1.487**	**0.308**	**0.002**	**0.112/0.504**
Self-employed	−0.069	0.625	−0.346/0.208	**0.998**	**0.000**	**0.498/1.498**	0.087	0.598	−0.237/0.411
Civil servant	0.087	0.497	−0.163/0.336	**0.949**	**0.000**	**0.587/1.312**	**0.305**	**0.005**	**0.093/0.516**
Retirement/Pension	0.080	0.683	−0.304/0.463	**1.124**	**0.002**	**0.428/1.820**	0.269	0.170	−0.116/0.655
**Household types**									
Living alone	0.056	0.664	−0.196/0.307	**0.760**	**0.000**	**0.389/1.132**	0.171	0.117	−0.043/0.386
With partner	−0.014	0.913	−0.269/0.241	**0.810**	**0.000**	**0.415/1.204**	0.125	0.253	−0.089/0.338
2 generation family (underage children)	−0.023	0.861	−0.281/0.235	**0.834**	**0.000**	**0.421/1.248**	0.141	0.235	−0.092/0.373
3 generation family	0.240	0.175	−0.107/0.587	**1.281**	**0.000**	**0.581/1.980**	**0.594**	**0.010**	**0.145/1.042**
1 generation shared flat	0.022	0.884	−0.270/0.313	**0.900**	**0.000**	**0.453/1.348**	0.047	0.704	−0.197/0.292
2 generation shared flat	0.247	0.284	−0.205/0.699	**1.238**	**0.003**	**0.420/2.057**	0.269	0.255	−0.194/0.732
Other types	−0.075	0.601	−0.356/0.206	**0.989**	**0.000**	**0.501/1.477**	0.050	0.699	−0.204/0.305
**Living environment**									
Rural area	0.041	0.737	−0.196/0.278	**0.801**	**0.000**	**0.437/1.164**	0.164	0.102	−0.033/0.361
Peri urban area	0.162	0.216	−0.095/0.420	**0.905**	**0.000**	**0.471/1.340**	**0.256**	**0.031**	**0.024/0.489**
Small town (<1 h from farmland)	0.163	0.211	−0.092/0.419	**0.947**	**0.000**	**0.498/1.395**	**0.351**	**0.002**	**0.124/0.578**
Small town (1–4 h from farmland)	0.106	0.487	−0.192/0.403	**1.073**	**0.000**	**0.573/1.572**	**0.562**	**0.000**	**0.270/0.853**
Big town (<4 h from farmland)	0.007	0.958	−0.263/0.278	**0.762**	**0.004**	**0.238/1.286**	0.048	0.791	−0.304/0.399
Big town (province capital)	0.134	0.373	−0.161/0.429	**0.858**	**0.000**	**0.423/1.293**	0.159	0.222	−0.096/0.413
City	0.287	0.090	−0.044/0.618	**1.563**	**0.000**	**0.879/2.247**	**0.435**	**0.010**	**0.105/0.765**
Mega city	−0.319	0.197	−0.804/0.166	**0.931**	**0.018**	**0.160/1.702**	−0.422	0.176	−1.034/0.189
Capital city	−0.009	0.949	−0.270/0.253	**0.727**	**0.005**	**0.217/1.236**	0.108	0.462	−0.181/0.397
	**Starchy vegetables**			**Legumes**			**Other vegetables**		
	**Mean difference**	***p***	**95% CI**	**Mean difference**	***p***	**95% CI**	**Mean difference**	***p***	**95% CI**
**Income regions**									
Low income	−0.087	0.825	−0.859/0.685	0.680	0.065	−0.043/1.404	0.582	0.341	−0.617/1.781
Lower middle income	**0.500**	**0.001**	**0.208/0.793**	**0.511**	**0.003**	**0.181/0.841**	**1.360**	**0.004**	**0.443/2.277**
Upper middle income	0.106	0.435	−0.160/0.371	0.399	0.077	−0.044/0.842	0.387	0.314	−0.366/1.140
High income	**0.182**	**0.007**	**0.049/0.316**	**0.478**	**0.032**	**0.040/0.916**	0.767	0.126	−0.216/1.750
**Gender**									
Female	0.188	0.168	−0.079/0.455	**0.558**	**0.004**	**0.179/0.938**	**0.883**	**0.036**	**0.059/1.708**
Male	0.151	0.242	−0.102/0.405	**0.554**	**0.001**	**0.231/0.878**	**0.700**	**0.031**	**0.064/1.335**
**Education**									
No degree/degree below level of	0.219	0.261	−0.163/0.600	**0.560**	**0.044**	**0.015/1.106**	1.038	0.080	−0.125/2.202
high school									
High school/A-level degree	0.002	0.991	−0.283/0.286	**0.524**	**0.003**	**0.183/0.865**	0.554	0.155	−0.210/1.317
Apprenticeship/vocational	0.265	0.093	−0.044/0.573	**0.414**	**0.019**	**0.069/0.759**	0.708	0.075	−0.072/1.487
baccalaureate diploma									
Vocational university diploma	0.187	0.121	−0.049/0.424	**0.754**	**0.000**	**0.402/1.106**	**0.856**	**0.011**	**0.198/1.515**
**Occupation**									
Student in school	0.212	0.352	−0.234/0.657	0.452	0.460	−0.755/1.659	−0.611	0.470	−2.269/1.048
University student/Trainee	0.180	0.110	−0.041/0.401	**0.459**	**0.004**	**0.145/0.773**	0.388	0.244	−0.265/1.040
Unemployed	0.354	0.168	−0.150/0.859	**1.126**	**0.003**	**0.396/1.856**	**1.562**	**0.025**	**0.200/2.924**
Employee	0.105	0.424	−0.152/0.361	**0.481**	**0.000**	**0.211/0.752**	**0.945**	**0.005**	**0.280/1.610**
Self-employed	0.039	0.868	−0.417/0.495	0.328	0.096	−0.058/0.714	**1.032**	**0.046**	**0.017/2.047**
Civil servant	0.082	0.552	−0.189/0.354	**0.437**	**0.001**	**0.175/0.699**	0.551	0.088	−0.083/1.186
Retirement/Pension	0.206	0.252	−0.147/0.558	**0.612**	**0.011**	**0.139/1.084**	**1.918**	**0.025**	**0.240/3.595**
**Household types**									
Living alone	0.175	0.154	−0.065/0.415	**0.488**	**0.005**	**0.149/0.827**	0.679	0.051	−0.003/1.361
With partner	0.112	0.430	−0.167/0.391	**0.575**	**0.002**	**0.213/0.936**	0.623	0.106	−0.132/1.378
2 generation family (underage children)	0.082	0.524	−0.170/0.333	**0.501**	**0.009**	**0.127/0.875**	0.464	0.218	−0.275/1.203
3 generation family	**0.508**	**0.024**	**0.067/0.949**	**0.604**	**0.004**	**0.195/1.013**	**1.589**	**0.017**	**0.284/2.895**
1 generation shared flat	0.041	0.842	−0.362/0.444	**0.482**	**0.023**	**0.065/0.898**	0.471	0.234	−0.305/1.248
2 generation shared flat	0.110	0.608	−0.311/0.531	**0.957**	**0.006**	**0.277/1.638**	1.328	0.067	−0.091/2.748
Other types	0.166	0.318	−0.160/0.491	0.326	0.091	−0.052/0.703	0.468	0.216	−0.273/1.210
**Living environment**									
Rural area	0.055	0.682	−0.207/0.317	**0.541**	**0.001**	**0.230/0.853**	0.620	0.084	−0.083/1.323
Peri urban area	0.167	0.258	−0.122/0.455	**0.689**	**0.001**	**0.273/1.105**	**0.866**	**0.025**	**0.109/1.622**
Small town (<1 h from farmland)	0.133	0.351	−0.146/0.412	**0.708**	**0.000**	**0.312/1.105**	**0.993**	**0.018**	**0.172/1.814**
Small town (1–4 h from farmland)	0.254	0.170	−0.109/0.616	**1.049**	**0.000**	**0.564/1.535**	**0.933**	**0.022**	**0.136/1.730**
Big town (<4 h from farmland)	0.214	0.253	−0.153/0.581	0.284	0.179	−0.130/0.698	0.621	0.211	−0.353/1.595
Big town (province capital)	0.056	0.697	−0.228/0.341	**0.485**	**0.016**	**0.092/0.877**	0.558	0.182	−0.262/1.377
City	0.210	0.192	−0.106/0.526	**0.834**	**0.002**	**0.310/1.358**	**1.294**	**0.010**	**0.316/2.271**
Mega city	0.224	0.263	−0.169/0.617	0.302	0.454	−0.488/1.092	0.488	0.595	−1.312/2.288
Capital city	0.216	0.239	−0.143/0.575	0.227	0.152	−0.084/0.537	0.621	0.130	−0.183/1.425

*Vegetable categories (max. 5), including dark green leafy vegetables, provitamin A rich vegetables, starchy vegetables, legumes and other vegetables, dark green leafy vegetables (max. 18), provitamin A rich vegetables (max. 8), Poisson regression, mean difference = mean before COVID-19—mean since COVID-19, thus, positive values indicate a reduction and negative values an increase in diversity over time; significance level: p <0.05, 95% CI = 95% confidence intervals, adjusted for all other tested predictors and age*.

*Starchy vegetables (max. 9), legumes (max. 17), and other vegetables (max. 33)*.

*Poisson regression, mean difference = mean before COVID-19—mean since COVID-19, thus, positive values indicate a reduction and negative values an increase in diversity over time; significance level: p < 0.05, 95% CI = 95% confidence intervals, adjusted for all other tested predictors and age*.

### Price Models

Poisson models that examined the effect of perceived price changes on the diversity of vegetable consumption showed no significant association for dark green leafy, starchy vegetables, legumes, and other vegetables. For provitamin A rich vegetables as well as for the diversity of the vegetable groups, a significant correlation was found with a negative coefficient of −0.011 and −0.006, respectively. The odds ratio showed that with a one unit increase in the price index, the chance of consuming a greater number of different provitamin A rich vegetables decreases by 1.1% when adjusted for age, gender, and income region (adOR = 0.989, 95% CI [0.980, 0.999], *p* = 0.029). In the case of overall vegetable diversity persons reporting a stronger increase in prices or increased prices in more food groups were more likely to cover less vegetable groups in their diet; one unit increase in the price index reduced the chance of consuming a larger number of vegetable groups by 0.6% (adOR = 0.994, 95% CI [0.989, 0.999], *p* = 0.024). Binary logistic regression models did not show significant correlations between price changes and increase or decrease of food consumption in general but only for change in vegetable consumption with a small but positive coefficient of 0.039. This indicates that the stronger the increase in perceived prices or the more food groups were affected by a rise in prices the more likely was a change in vegetable consumption. With a one unit increase in the price index, the chance of changing one's vegetable consumption increased by 4% (adOR = 1.040, 95% CI [1.014, 1.067], *p* = 0.003; Table 5).

**Table 5:** Results of binary logistic and Poisson regressions for the independent variable “perceived price changes”^*^.

	**Coefficient**	***p***	**OR**	**95% CI lower bound**	**95% CI upper bound**
Decrease in food quantity^§^	0.022	0.280	1.022	0.982	1.064
Increase in food quantity^§^	0.015	0.656	1.015	0.950	1.084
Vegetable consumption^§^	**0.039**	**0.003**	**1.040**	**1.014**	**1.067**
Vegetable categories^#^	**−0.006**	**0.024**	**0.994**	**0.989**	**0.999**
Dark green leafy vegetables^#^	−0.007	0.202	0.993	0.981	1.004
Provitamin A rich vegetables^#^	**−0.011**	**0.029**	**0.989**	**0.980**	**0.999**
Starchy vegetables^#^	−0.014	0.062	0.986	0.972	1.001
Legumes^#^	−0.012	0.067	0.988	0.975	1.001
Other vegetables^#^	−0.008	0.195	0.993	0.981	1.004

*Vegetable categories = dark green leafy vegetables, provitamin A rich vegetables, starchy vegetables, legumes, and other vegetables*.

* *The changes in prices for all food groups were summed up with 2 points for a “strong increase,” 1 point for a “little increase,” 0 points for “no change,” −1 points for a “little decrease,” and −2 points for a “strong decrease” per food group*.

§ *Binary logistic regression (food quantity, vegetables consumption)*.

# *Poisson regression (vegetable categories), OR, odds ratio, significance level: p < 0.05, 95% CI = 95% confidence intervals, adjusted for age, gender, and income regions*.

## Discussion

In this study, one out of five persons ate more than prior to the Pandemic whereas fewer people reported to eat less. At the same time, the findings of this study showed that the restrictions and lockdown events negatively impacted on the level of diversity in vegetable consumption. The reduced consumption of different vegetable types was only partly due to lockdown scenarios but mainly due to individual factors which became probably more pronounced by the side effects of the pandemic.

### Changes in Food Quantity

Decreased appetite or feeling of hunger, lower caloric needs due to less physical effort, losing, or stabilising weight, mental stress, reduction of out of home consumption, and price increases were described as the reasons of a reduction of quantity of food consumed since the pandemic started. Besides general reasons for controlling one's eating habits such as the caloric intake, most of the given reasons were related to the implemented restrictions. Overall, the reasons given for the reduction in food quantity were more diverse than the ones for the increase since the onset of the pandemic.

After the pandemic has been declared, 22.9% of the respondents reported to have consumed more food and 15.1% less food. This rate was lower than in a Polish study which showed that the proportion of people eating more than before COVID-19 was 43.5%—almost twice as high as in this study (30). An online survey among 1,964 Bavarian university students in March/April 2020 also reported higher levels for an increase in food intake, i.e., 31.2% reporting an increase and 16.8% a decrease during the lockdown (31). Whereas, a Dutch study conducted in April 2020 with 1,030 participants reported much lower rates for both directions of change; 8.2% ate less and 8.9% more food during lockdown (32) which was even lower than in our study. A survey conducted among 879 adults with a mean age of 36 years in Saudi Arabia in late April 2020 showed that the majority (57.5%) changed the number of meals during the day during the curfew in comparison to the meals before COVID-19 which indicates a change in the amount of food intake, too (33). However, all these studies observed only a period of 1–2 weeks during the very beginning of the pandemic in March/ April 2020 whereas in our study we observed a period of more than 4 months. On average, it was more likely to eat more than less food following the declaration of the pandemic which lasted even if there was no lockdown or restriction anymore as could be seen in this study. This shows that changes in dietary behaviour were not just a short-term effect at the beginning of the pandemic but lasted much longer. Still, these data may have flawed along the line of the respective restrictions put in place.

Food intake changes were not associated with differences between lockdown scenarios or specific restrictions. This might be related to one's mental state, personal coping strategies, and individual reaction to governmental regulations (34). The reaction on the restrictions on a personal level might be more important in this context than the specific restrictions themselves.

A significant effect of age on food intake was found in our study with respect to increase in food quantity. The younger the participants were, the more likely they reported an increase in the amount of food they had eaten since the COVID-19 pandemic. Results of the Bavarian study mentioned above also indicated that younger people more likely changed the amount of food they consumed (31). This may be explained with that younger persons are less resilient toward crisis like this pandemic and thus more prone to stress (35). The increased food intake is considered to be a compensation strategy for stress or to comfort themselves (36). Furthermore, older individuals might be less affected by emotional eating and thus have a more stable dietary behaviour (35, 37). However, unlike our results there was no effect for age in the Polish population under quarantine (30).

Similar to Sidor and Rzymski (30) we could not detect any significant effects for gender, educational level, occupation, or place of living on overall food intake changes. Emotional eating and depression is linked to each other and may be moderated by gender, thus, women showing stronger effects than men (36, 38). In our study being a woman was not associated with overall food intake changes but with a higher chance to eat less diverse. However, a study in the Netherlands showed that women were more likely to eat more during a lockdown compared to men and that participants within the group of lower educational level were more likely to have reduced their food intake since the beginning of the lockdown (32) which could not be confirmed in this study.

A multi-country study conducted from mid-April to end of May using the same method as in this study showed a strong relation between country of residence and the mean food intake since the onset of the pandemic (39). Because of the imbalanced sample we did not test the effect of different countries. However, no significant influence of income regions on the change of food quantity was found in our sample. Evaluating the influence of perceived changes in food prices our findings showed no significant correlation, neither with decrease nor with increase in overall food quantity consumed.

The hypothesis that mental stress and anxious feelings could be one reason for a change in food quantity was supported by the study of Di Renzo et al. which showed that anxious feelings were likely to occur during the pandemic due to isolation (36). Furthermore, their respondents declared eating more to comfort themselves. The occurrence of over-eating since the lockdown was more noticeable in individuals who were older, had a higher BMI, were not on a diet before COVID-19 and who felt anxious since the COVID19 outbreak (36, 40). Emotional eating was more likely to occur to persons with a higher BMI, with more symptoms of a depression, and higher levels of anxiety (41). The same study supports the hypothesis that loss of life quality due to the lockdown and psychological distress may cause an increase in perceived emotional eating (41) which was not looked at in this study.

### Changes in Overall Vegetable Consumption

Any change in overall vegetable consumption was reported by 27% of the participants in this study. Almost the same number of persons stated to have increased their vegetable intake to those who reported a reduction since the beginning of the COVID-19 pandemic. Moreover, a self-reported shift from fresh and perishable vegetables toward canned, frozen, and storable vegetables emerged. Reasons given for the decline in vegetable intake included reduced availability and access, rise in prices, reduced shopping frequency, seasonality, and changes in work situations. In the case of the increase in vegetable consumption, reasons mentioned by the respondents included more home-cooked meals, for better health and immunity, more time to cook, seasonality, switching to a vegetarian diet and for a higher variation of meals. Vegetable intake is associated with habit, motivation, knowledge, and goals (42). This indicates that individual decisions may play a greater role than social groups in changing vegetable consumption during the pandemic.

Whereas, agrobiodiversity loss has already caused production losses and food insecurity, the current Covid-19 pandemic and related food crisis has in addition contributed to an increase in food insecurity (43, 44) and the consumption of mainly perishable foods such as fresh fruits and vegetables, meat and dairy declined (45). An overall change or a change in either one or the other direction in vegetable consumption was also seen in other studies. A large consumer study in Denmark, Germany and Slovenia observed in the very beginning of the lockdown about the same prevalence of change in vegetable consumption with more respondents reporting a decrease (15.2–22.6%) than increase (7.3–12.0%) (46). In the same study a shift from fresh foods toward foods with longer shelf life was observed, too (46). Young people from Southern Europe and South America had significantly increased the consumption of vegetables and legumes (39). In the multi-country study the proportion of adolescents who consumed the recommended weekly amount of legumes (2–4 servings) even increased and 7.8% more young people ate vegetables every day (35% before COVID-19 to 43% during confinement) (39). A survey from Spain also showed an increase in the consumption of legumes during confinement. In this case, the number of subjects who stated that they ate at least 3 portions of pulses a week increased by 6.1% from 25.4 to 31.5% (47). In contrast, a decrease in the frequency of legumes consumption has been observed for Ethiopia since the beginning of the COVID-19 pandemic and 22% of the respondents reported, vegetables were no longer consumed due to rumours that certain foods could lead to COVID-19 infection (48). Other studies also showed a decline in the consumption of vegetables and fresh fruits (8, 30). In Iran the greatest change over time was observed for white roots and dark green leafy vegetables whereas provitamin A rich vegetables were the most consumed vegetables in these households both before and since the COVID-19 outbreak (8). Whereas, in Bavaria, Germany, no relevant difference in the consumption of fruits and vegetables among members of Bavarian universities was observed (31).

The evaluation of all potential factors influencing the change in vegetable consumption showed no significant correlations in our models. In contrast, Ruiz-Roso et al. (39) observed significantly higher intakes of fruits and vegetables by girls than by boys during confinement. They also reported that adolescents from households with at least seven members were the least likely to meet weekly vegetable intake recommendations in comparison to all household groups with fewer members (39). The same study compared different countries and showed that in Southern Europe and South America, Colombia had the lowest rates of vegetable consumption, while Brazil was the country with the highest legume consumption. Spain, on the other hand, was the only one of the countries studied that did not show an increase in legume intake since the beginning of the pandemic (39). It seems that age plays a role as a significant increase in vegetable intake was only detected for adolescents over 14 years of age (39) whereas individuals over 45 years of age were to be the ones with the lowest frequency of daily fruit and vegetable intake (63%) and daily intake of legumes (15.3%) in Poland (30).

Being a woman was indicated to be a risk factor toward feeling challenged to eat healthy foods, while older respondents were more likely to face no such obstacles (32). These findings suggest that commonly accepted determinants for poor dietary choices were reinforced during the pandemic.

### Changes in Vegetable Diversity

To date, no comparable studies are available that address the changes of vegetable diversity due to the COVID-19 pandemic. A study in the United States using data from a digital behaviour change weight loss program observed a decrease in the consumption of salads while the consumption of starchy vegetables increased, which indicates a shift in vegetable selection but not whether less vegetable types were consumed (49). In general, higher diversity of vegetables can lead to the intake of a larger range of vitamins, minerals, and phytochemicals, which in turn can have a positive effect on health and nutritional status (50, 51). For most restrictions types and lockdown scenarios we could not identify any effect on the diversity of vegetable consumption. Only curfew at night was positively associated with an increase in legume diversity. The fact that lockdown scenarios and restrictions did mostly show no effects on overall vegetable consumption might be a result of globalised trade. Barriers and bans installed by some countries or regions may have led to unavailability of specific vegetables in countries not affected by a lockdown themselves (52). For example, the lockdown in Spain and Italy could have led to a limitation of vegetables in the European market due to their important role as vegetable producers and exporters (13). This effect may have occurred in other regions of the world as well yet, might have been compensated by the countries own production not being exported anymore. Contact and travel restrictions implemented by certain countries have led to issues in the harvesting and transport sectors because of border closures and lack of field workers that normally come from abroad (53). Countries depending on vegetable imports may have faced issues in providing their population with a high diversity of vegetables even though they did not implement restrictions themselves (52). In this context, lockdowns implemented in certain countries may have had an impact on global trade and availability of vegetable diversity (54) but this could not been shown within this study.

To identify potential vulnerable groups, we tested changes in vegetable diversity over time for different social groups and for different living environments. Our results suggest that the region where people reported from, the “income regions,” played a crucial role for diversity of all vegetable groups and the overall diversity consumed in both time periods. The same effect was observed for gender except for the starchy vegetables and legumes. Household types had a significant effect on the overall diversity prior to COVID-19 and on the category other vegetables for both time periods. The fact that in several cases pre-COVID-19 effects disappeared since the COVID-19 outbreak indicates that the food environment has converged between the different groups. This may reflect that overall supply and availability were important factors but also that individuals had in most cases fewer opportunities for out of home eating than before. Moreover, the change may be caused by more than one predictor, as especially in the global context it is likely that potential reasons differ in certain regions. However, this would need to be confirmed in further studies.

### Change of Food Prices and Vegetable Consumption

Our findings showed that perceived changes in food prices are significantly correlated with the change in vegetable consumption. The stronger the increase in perceived prices or the more food groups were affected by a rise in prices, the more likely was a change in vegetable consumption. An increase in prices can lead to issues in affordability, especially in combination with loss of income (55). Due to consumer decisions this may affect the supply of vegetable more than the supply of staple foods.

In the case of perceived price changes for a basic food basket, our study showed that there was a significant association with the number of vegetable groups consumed and the number of different provitamin A rich vegetable types. Within all other vegetable groups, no effect of price changes on the variety was observed which maybe also due to only about 5% of respondents experiencing a loss of their job. Due to increased prices, especially in combination with loss of income, respondents may have had to compromise on their vegetable diversity (55). The difference between the provitamin A rich vegetables and the four other vegetable groups might be the result of a different extent of price rises for the different vegetable groups or a different impact on the availability due to seasonality and trade restrictions as was mentioned by a German respondent in the open answers. The latter showed also that the less frequent shopping, worsening of food availability in stores, closures of canteens, no motivation or time for cooking, and seasonality in the context of certain countries contributed to the observed change in vegetable consumption patterns. Personal situations including time availability, mental state, and motivation to cook and diversify the diet could have played a major role as shown by the open answers in our study. This may also be an explanation for the lack of differences between restriction scenarios.

## Summary and Conclusion

In our international survey on Food and COVID-19 more increase than decrease of general food consumption was detected from April to July 2021 compared to the period prior to the pandemic. The reaction on the COVID-19 restrictions on a personal level were more decisive influencing food consumption than the specific restrictions themselves. The increase in vegetable consumption was reported by as many participants as the decrease and a clear shift from fresh and perishable vegetables toward canned, frozen, and storable vegetables was observed. The restrictions and lockdown events negatively impacted the diversity in vegetable consumption but mainly due to individual factors which became probably more pronounced by the side effects of the pandemic. The most vulnerable to greatest reduction in diversity in vegetable consumption were those living in lower middle income countries, being a woman, having a university degree, being unemployed, living in a 3-generational family and living in a small town. Perceived changes in food prices were significantly correlated with the change in vegetable consumption. The stronger the increase in perceived prices or the more food groups were affected by a rise in prices, the more likely was a change in vegetable consumption.

Food systems are not static and are transitioning quickly as could be observed during the Covid-19 pandemic. Consequently, a nutrition strategy is needed to strengthen the resilience of all households so that they can consume a balanced, diverse, and sustainable diet in sufficient quantities especially as regards highly perishable foods such as vegetables for planetary health (20).

## Strengths and Limitations

The strength of this study are the sample size and the internationality of the study participants. This enabled us to provide a first overview about the impact of the Covid-19 pandemic at international level who responded to the same questions although the number of respondents from low income countries was limited. The latter are shown to complete the picture, yet, should be used with care. We also have to acknowledge that the chosen method, online survey, is a barrier for participation from most vulnerable populations, poor people, and/or elders who do not have access to the resources. Also, it was reported to us that the poor internet capacities in some countries hindered people to participate. Therefore, results should be interpreted with caution only, especially for the low income countries. Nevertheless, we think that our results can contribute to the ongoing debate on dietary diversity and serve as initial estimates that should be followed up by conducting representative studies.

The survey covered an important time during the first half year of the pandemic and allowed to observe different scenarios of restrictions. At the same time the long period may have biassed the recall of the participants in terms of dietary patterns prior to the lockdown. Like with food frequency questionnaires underestimation can be expected (56). This study did not randomly select participants, but relied on volunteers who may have participated because they were more health conscious. This may have limited the recall bias. In this study we used an explorative approach and due to the lockdown and mobility restrictions designed it as online survey. We did not have funds available to facilitate the data collection with a company support and/or telephone-based interviews which limited our possibilities to mobilise a larger number of respondents. However, we think the sample size allows to get a first impression how the pandemic has impacted consumers at global level. The results serve thus to generate and not to confirm hypotheses on how Covid-19 impacted dietary intake of populations.

Despite the limitations of the study, this study is the first to look at the diversity of food intake at global level and the findings show that there is an urgent need to pay attention to vegetable diversity in local and global food systems and in research on the same.

## Data Availability Statement

The raw data supporting the conclusions of this article will be made available by the authors, without undue reservation.

## Ethics Statement

The studies involving human participants were reviewed and approved by Review Board of the medical faculty of the Justus Liebig University Gießen, Germany. The patients/participants provided their written informed consent to participate in this study.

## Author Contributions

LS conducted the data cleaning and the statistical analysis under the lead of IJ. IJ prepared the manuscript based on the findings from LS with contributions from GK, KJ, IH, and EH. IJ, LS, KJ, IH, and EH developed and translated the questionnaire with the support of an international network. IJ was the principle investigator and responsible for the conceptualisation of the study design. All the authors read and approved the final manuscript.

## Conflict of Interest

The authors declare that the research was conducted in the absence of any commercial or financial relationships that could be construed as a potential conflict of interest.

## Publisher's Note

All claims expressed in this article are solely those of the authors and do not necessarily represent those of their affiliated organizations, or those of the publisher, the editors and the reviewers. Any product that may be evaluated in this article, or claim that may be made by its manufacturer, is not guaranteed or endorsed by the publisher.
