# Supplementary material for: Changes in Vegetable Consumption in Times of COVID-19—First Findings From an International Civil Science Project
Source: Front Nutr. 2021 Aug 17;8:686786. doi: 10.3389/fnut.2021.686786 (PMC8415868; doi:10.3389/fnut.2021.686786)
Supplement: Supplementary file 1 [file Data_Sheet_1.docx]

Supplementary material

Table 1: National distribution of responses stratified by income region according to World Bank classification (1)

| Low income countries (n=16) | n | Lower middle income countries (n=123) | n | Upper middle income countries (n=94) | n | High income countries (n=804) | n |
| --- | --- | --- | --- | --- | --- | --- | --- |
| 1. **Ethiopia** | 10 | 1. **Bangladesh** | 2 | 1. Albania | 2 | 1. Australia | 3 |
| 1. Gambia | 2 | 1. Bolivia | 2 | 1. Argentina | 3 | 1. Austria | 5 |
| 1. Somalia | 1 | 1. Cambodia | 1 | 1. Brazil | 2 | 1. Belgium | 1 |
| 1. Uganda | 3 | 1. Benin | 2 | 1. **China (People`s Republic of China)** | 38 | 1. Canada | 3 |
|  |  | 1. Ghana | 5 | 1. Colombia | 14 | 1. Chile | 1 |
|  |  | 1. Honduras | 3 | 1. Costa Rica | 2 | 1. Croatia | 1 |
|  |  | 1. India | 6 | 1. **Ecuador** | 10 | 1. Czech Republic | 1 |
|  |  | 1. **Kenya** | 13 | 1. Fiji | 2 | 1. Denmark | 1 |
|  |  | 1. Morocco | 1 | 1. Georgia | 1 | 1. **Germany** | 693 |
|  |  | 1. **Nigeria** | 3 | 1. Guatemala | 2 | 1. Greece | 2 |
|  |  | 1. **Vietnam** | 83 | 1. Indonesia | 3 | 1. Hong Kong | 1 |
|  |  | 1. Zimbabwe | 1 | 1. Kazakhstan | 1 | 1. Ireland | 2 |
|  |  | 1. Tanzania | 1 | 1. México | 2 | 1. **Italia** | 6 |
|  |  |  |  | 1. Namibia | 1 | 1. Japan | 4 |
|  |  |  |  | 1. Peru | 4 | 1. South Korea | 1 |
|  |  |  |  | 1. Russia | 1 | 1. Netherlands | 4 |
|  |  |  |  | 1. St. Vincent and the Grenadines | 1 | 1. New Zealand | 2 |
|  |  |  |  | 1. South Africa | 1 | 1. Norway | 1 |
|  |  |  |  | 1. Taiwan | 3 | 1. **Poland** | 13 |
|  |  |  |  | 1. Venezuela | 1 | 1. Singapore | 1 |
|  |  |  |  |  |  | 1. **Spain** | 7 |
|  |  |  |  |  |  | 1. Sweden | 1 |
|  |  |  |  |  |  | 1. Switzerland | 8 |
|  |  |  |  |  |  | 1. United Kingdom | 6 |
|  |  |  |  |  |  | 1. **United States** | 36 |
| The countries marked in bold indicate the countries of origin of the partners who were actively involved in the questionnaire development, translation and dissemination (n=12). | | | | | | | |

(1) The World Bank. World Bank Country and Lending Groups – World Bank Data Help Desk [Internet]. 2021 [cited 2021 Mar 9]. Available from: https://datahelpdesk.worldbank.org/knowledgebase/articles/906519-world-bank-country-and-lending-groups

Table 2: Results of the binary logistic regressions for the independent variable “age”

|  | Coefficient | p-value | OR | 95% CI lower bound | 95% CI upper bound |
| --- | --- | --- | --- | --- | --- |
| Decrease in food quantity | -0.017 | 0.747 | 0.983 | 0.888 | 1.089 |
| Increase in food quantity | **-0.101** | **0.018** | **0.904** | **0.831** | **0.983** |
| Vegetable consumption | -0.064 | 0.103 | 0.938 | 0.868 | 1.013 |
| Vegetable categories | 0.003 | 0.386 | 1.003 | 0.997 | 1.009 |
| Vegetable categories*Time | -0.002 | 0.384 | 0.998 | 0.993 | 1.003 |
| Dark green leafy vegetables | 0.009 | 0.337 | 1.009 | 0.990 | 1.029 |
| Dark green leafy vegetables*Time | 0.015 | 0.058 | 1.015 | 0.999 | 1.030 |
| Vitamin A rich vegetables | 0.008 | 0.351 | 1.008 | 0.992 | 1.024 |
| Vitamin A rich vegetables*Time | 0.005 | 0.460 | 1.005 | 0.992 | 1.018 |
| Starchy vegetables | 0.021 | 0.118 | 1.022 | 0.995 | 1.049 |
| Starchy vegetables*Time | 0.001 | 0.917 | 1.001 | 0.980 | 1.023 |
| Legumes | -0.006 | 0.671 | 0.994 | 0.969 | 1.020 |
| Legumes*Time | 0.013 | 0.151 | 1.013 | 0.995 | 1.032 |
| Other vegetables | 0.005 | 0.597 | 1.005 | 0.985 | 1.026 |
| Other vegetables*Time | **0.018** | **0.016** | **1.018** | **1.003** | **1.033** |
| Vegetable categories=dark green leafy vegetables, vitamin A rich vegetables, starchy vegetables, legumes, and other vegetables  Time= prior to and after pandemic was declared  Binary logistic regression (food quantity and vegetables consumption), Poisson regression (vegetable categories), OR=Odds ratio, Significance level: p<0.05, 95% CI=95% Confidence intervals,  adjusted for gender, income region, occupation, education, household types and living environment | | | | | |

Table 3: Perceived number of vegetables consumed within each category before and since the beginning of the COVID-19 pandemic in relation to lockdown scenarios and income region

|  | **Dark green leafy vegetables** | | **Vitamin A rich vegetables** | | **Starchy vegetables** | | **Legumes** | | **Other vegetables** | |  |
| --- | --- | --- | --- | --- | --- | --- | --- | --- | --- | --- | --- |
| Covid-19-> | Before | Since | Before | Since | Before | Since | Before | Since | Before | Since |  |
| **No lockdown (n=215)** | | | | | | | | | | | |
| maximum* | 18 | 12 | 8 | 8 | 8 | 4 | 11 | 10 | 21 | 20 |  |
| 25^th^ percentile | 2 | 1 | 2 | 1 | 0 | 0 | 1 | 1 | 3 | 2 |  |
| median | 3 | 3 | 3 | 2 | 1 | 1 | 3 | 2 | 8 | 8 |  |
| 75^th^ percentile | 5 | 5 | 4 | 3 | 1 | 1 | 4 | 4 | 12 | 12 |  |
| **No lockdown anymore (n=163)** | | | | | | | | | | | |
| maximum* | 18 | 12 | 7 | 7 | 5 | 6 | 15 | 9 | 23 | 23 |  |
| 25^th^ percentile | 2 | 2 | 1 | 1 | 0 | 0 | 1 | 0 | 2 | 1 |  |
| median | 4 | 3 | 3 | 2 | 1 | 1 | 2 | 2 | 9 | 8 |  |
| 75^th^ percentile | 6 | 5 | 4 | 3 | 2 | 2 | 5 | 4 | 14 | 13 |  |
| **Lockdown (n=655)** | | | | | | | | | | | |
| maximum* | 17 | 17 | 8 | 8 | 9 | 8 | 17 | 15 | 33 | 31 |  |
| 25^th^ percentile | 3 | 2 | 2 | 2 | 0 | 0 | 1 | 1 | 5 | 4 |  |
| median | 4 | 3 | 3 | 3 | 1 | 1 | 3 | 2 | 9 | 9 |  |
| 75^th^ percentile | 6 | 5 | 4 | 4 | 2 | 1 | 5 | 5 | 13 | 12 |  |
| **Low-income countries (n=16)** | | | | | | | | | | | |
| maximum* | 12 | 6 | 8 | 7 | 5 | 5 | 10 | 6 | 15 | 10 |  |
| 25^th^ percentile | 0.25 | 0 | 0.25 | 0 | 0.25 | 0 | 0 | 0 | 1.25 | 0.25 |  |
| median | 2 | 1 | 1 | 1 | 1 | 1 | 2 | 1 | 3 | 2 |  |
| 75^th^ percentile | 2 | 1.75 | 2 | 2 | 1.75 | 1 | 3.75 | 2 | 5.75 | 5 |  |
| **Lower-middle income countries (n=123)** | | | | | | | | | | | |
| maximum* | 18 | 17 | 8 | 7 | 9 | 8 | 15 | 14 | 33 | 31 |  |
| 25^th^ percentile | 1 | 0 | 0 | 0 | 0 | 0 | 0 | 0 | 1 | 1 |  |
| median | 3 | 2 | 2 | 1 | 1 | 1 | 1 | 1 | 5 | 3 |  |
| 75^th^ percentile | 5 | 3 | 3 | 3 | 3 | 2 | 2 | 1 | 10 | 7 |  |
| **Upper-middle income countries (n=94)** | | | | | | | | | | | |
| maximum* | 13 | 15 | 6 | 6 | 6 | 7 | 9 | 9 | 18 | 18 |  |
| 25^th^ percentile | 1 | 1 | 1 | 0.75 | 0 | 0 | 1 | 0 | 1 | 1 |  |
| median | 3 | 2 | 3 | 2 | 2 | 1 | 2 | 2 | 3.5 | 3.5 |  |
| 75^th^ percentile | 5 | 4 | 4 | 3 | 3 | 3 | 4 | 4 | 9.25 | 8.25 |  |
| **High-income countries (n=804)** | | | | | | | | | | | |
| maximum* | 13 | 12 | 8 | 8 | 5 | 4 | 17 | 15 | 28 | 24 |  |
| 25^th^ percentile | 3 | 2 | 2 | 2 | 0 | 0 | 2 | 1 | 6 | 6 |  |
| median | 4 | 4 | 3 | 3 | 1 | 1 | 3 | 3 | 10 | 10 |  |
| 75^th^ percentile | 6 | 5 | 4 | 4 | 1 | 1 | 5 | 5 | 13.25 | 13 |  |
| *=minimum was in all cases = 0 | | | | | | | | | | |  |
